# β2-adrenergic stress evaluation of coronary endothelial-dependent vasodilator function in mice using ^11^C-acetate micro-PET imaging of myocardial blood flow and oxidative metabolism

**DOI:** 10.1186/s13550-014-0068-9

**Published:** 2014-12-16

**Authors:** Etienne Croteau, Jennifer M Renaud, Christine Archer, Ran Klein, Jean N DaSilva, Terrence D Ruddy, Rob SB Beanlands, Robert A deKemp

**Affiliations:** National Cardiac PET Centre, Department of Medicine, Division of Cardiology, University of Ottawa Heart Institute, 40 Ruskin Street, Ottawa, K1Y 4W7 ON Canada

**Keywords:** Endothelial function, Adrenergic receptor, Vasodilatation, Vascular reactivity sympathetic nervous system, Myocardial blood flow

## Abstract

**Background:**

Endothelial dysfunction is associated with vascular risk factors such as dyslipidemia, hypertension, and diabetes, leading to coronary atherosclerosis. Sympathetic stress using cold-pressor testing (CPT) has been used to measure coronary endothelial function in humans with positron emission tomography (PET) myocardial blood flow (MBF) imaging, but is not practical in small animal models. This study characterized coronary vasomotor function in mice with [^11^C]acetate micro-PET measurements of nitric-oxide-mediated endothelial flow reserve (EFR_NOM_) (adrenergic-stress/rest MBF) and myocardial oxygen consumption (MVO_2_) using salbutamol β_2_-adrenergic-activation.

**Methods:**

[^11^C]acetate PET MBF was performed at rest + salbutamol (SB 0.2, 1.0 μg/kg/min) and norepinephrine (NE 3.2 μg/kg/min) stress to measure an index of MBF response. β-adrenergic specificity of NE was evaluated by pretreatment with α-adrenergic-antagonist phentolamine (PHE), and β_2_-selectivity was assessed using SB.

**Results:**

Adjusting for changes in heart rate × systolic blood pressure product (RPP), the same stress/rest MBF ratio of 1.4 was measured using low-dose SB and NE in normal mice (equivalent to human CPT response). The MBF response was correlated with changes in MVO_2_ (*p* = 0.02). Nitric oxide synthase (NOS)-inhibited mice (N^g^-nitro-L-arginine methyl ester (L-NAME) pretreatment and endothelial nitric oxide synthase (eNOS) knockout) were used to assess the EFR_NOM_, in which the low-dose SB- and NE-stress MBF responses were completely blocked (*p* = 0.02). With high-dose SB-stress, the MBF ratio was reduced by 0.4 following NOS inhibition (*p* = 0.03).

**Conclusions:**

Low-dose salbutamol β_2_-adrenergic-stress [^11^C]acetate micro-PET imaging can be used to measure coronary-specific EFR_NOM_ in mice and may be suitable for assessment of endothelial dysfunction in small animal models of disease and evaluation of new therapies.

**Electronic supplementary material:**

The online version of this article (doi:10.1186/s13550-014-0068-9) contains supplementary material, which is available to authorized users.

## Background

Coronary artery disease (CAD) is a leading cause of death worldwide, and endothelial dysfunction (ED) is an early marker in the progression of coronary atherosclerosis. The principal function of the coronary endothelium is auto-regulation of microvascular perfusion in response to the changing metabolic demands of the heart [1]. Assessment of coronary endothelial function is important as ED can accelerate the progression of several cardiac-related diseases, including hypertension, diabetes, and atherosclerosis [2]. With advanced CAD, endothelial vasodilatation can be severely inhibited, or paradoxically, vasoconstriction can occur in response to metabolic or sympathetic stress [3].

Limited *in vivo* methods are available to investigate ED: 1) invasive catheterization of epicardial coronary arteries with Doppler ultrasound flow response to intracoronary infusion of acetylcholine (ACh) or salbutamol (SB) [3],[4], 2) non-invasive echocardiography measurement of large peripheral arteries with flow-mediated dilation (FMD) response to shear stress [5], and 3) non-invasive myocardial blood flow (MBF) positron emission tomography (PET) sympathetic-stress response to cold-pressor testing (CPT) [6],[7]. Immersion of the hand or foot in ice water for CPT predominantly releases the catecholamine norepinephrine and increases systemic blood pressure and heart rate. The correspondingly higher heart rate × systolic blood pressure product (RPP) is an index of cardiac work correlated to an increase of myocardial oxygen consumption [8].

In angiographically normal human coronary arteries, the non-invasive PET CPT flow increase is highly correlated with the intracoronary Doppler flow response to ACh infusion [9], whereas patients with abnormal CPT MBF response (<40%) have increased risk of clinical cardiovascular events [10]. The CPT response is predominantly adrenergic-receptor mediated and endothelium-dependent [11],[12], representing only a fraction of the maximal vasodilatation usually assessed with pharmacologic stressors that directly target adenosine receptors/reuptake at the coronary vascular smooth muscle [13].

The PET perfusion tracers [^13^ N]ammonia and [^15^O]water have been used to quantify MBF in rats [14],[15]; however, their long positron ranges (RMS approximately 0.6 and 1.0 mm, respectively) with adjacent liver and lung uptake are not ideal characteristics for PET imaging of the approximately 1-mm thickness myocardium in mice [16]. [^11^C]acetate has a shorter positron range (0.4 mm), minimal liver and lung uptake in mice, and a washout rate (*k*_2_) which is accepted as an index of oxidative metabolism in human and animal studies [15]. Additionally, the tracer uptake rate (*K*_1_) has been proposed as an index of MBF and used to derive absolute flow values in humans [17] and rats [15], making it an attractive candidate for PET imaging of MBF in mice.

The goal of this study was to assess the coronary-specific endothelium-dependent MBF response using micro-PET imaging with low-dose SB, as an alternative to sympathetic stress using norepinephrine (NE) or CPT PET imaging that is well-established in humans [6],[7]. SB is a selective β_2_-adrenergic agonist and potent bronchodilator, commonly administered in aerosol form to treat the symptoms of asthma. However, it is also known that the β_2_-adrenergic receptors in the heart are expressed mainly in the small arteries and precapillary arterioles and therefore may be an ideal target to selectively probe the vasodilator function of the coronary microvasculature [18]. If successful, SB-stress PET imaging has the potential for direct translation to human studies investigating the progression of endothelial dysfunction and the evaluation of new therapies.

## Methods

### Animal preparation

Normal adult male C57BL/6 J mice (6 to 10 weeks old; Charles River, Montreal, Canada) weighing 27 ± 4 g (*n* = 40) and adult male endothelial nitric oxide synthase knockout (eNOS^−/−^) 129S6.129P2(B6)-Nos3tm1Unc/J mice (6 to 8 weeks old; Jackson Laboratory, Bar Harbor, ME, USA) weighing 26 ± 6 g (*n* = 5) were included in this study.

### Physiologic monitoring

Mice were anesthetized with 1% to 2% isoflurane (Abbott Laboratories, Montreal, Canada) and monitored using external probes (SA Instruments Inc., Stony Brook, NY, USA). Venous catheters were inserted into one or both lateral tail veins for adrenergic stressor and PET radiotracer administration. Blood pressure was monitored with a pressure analyzer connected to an ultra-miniature fiber optic sensor (SA Instruments Inc., Stony Brook, NY, USA) attached to a PE10 polyethylene catheter (Becton Dickinson, Franklin Lakes, NJ, USA) inserted into the carotid artery. For validation of the PET radiotracer measurements in arterial blood (tracer kinetic input function), the same PE10 carotid catheter method was used for manual blood withdrawal (*n* = 5).

Three experimental groups were used (Table 1) with *n* = 5 each as follows: 1) normal control mice under SB-stress and NE-stress ± pretreatment with phentolamine (PHE), an α-adrenergic receptor antagonist; 2) NOS-inhibition mice pretreated using N^g^-nitro-L-arginine methyl ester (L-NAME) under SB-stress and NE-stress; and 3) transgenic knockout eNOS^−/−^ mice under NE-stress. The catecholamine NE was used at 1.6, 3.2, and 5.6 μg/kg/min (Hospira, Lake Forest, IL, USA) [19], phentolamine (Novartis, Quebec, Canada) at 1.5 mg/kg I.V. administered 5 min before NE-stress PET imaging [20], and SB was used at high and low doses of 1.0 and 0.2 μg/kg/min [21]. The middle dose of 3.2 μg/kg/min NE was selected as the reference standard (NE control). Pretreatment with L-NAME (Sigma-Aldrich, Oakville, Canada), a non-selective systemic nitric oxide synthase inhibitor, was administered at 40 to 60 mg/kg/day via drinking water for 1 week [22]. All animal experiments were performed in accordance with the Canadian Council on Animal Care guidelines and ethics approval by the University of Ottawa Animal Care Committee.

**Table 1:** **Experimental groups used for adrenergic stress characterization of coronary endothelial function**

Adrenergic stress agent	Receptor target	I.V. dose (μg/kg/min)	Cardiovascular effects	Normal mice (***n***)	NOS-inhibition mice (***n***)	eNOS-knockout mice (***n***)
NE	Alpha-agonist + beta-agonist	3.2	Alpha peripheral vasoconstriction beta_1_ cardiac inotropy and chronotropy beta_2_ coronary and bronchial dilatation	5	5	5
PHE + NE	Alpha-antagonist + beta-agonist	3.2	Alpha peripheral vasodilatation beta_1_ cardiac inotropy and chronotropy beta_2_ coronary and bronchial dilatation	5		
SB high-dose	Beta_2_-agonist	1.0	Beta_2_ coronary and bronchial dilatation	5	5	
SB low-dose	Beta_2_-agonist	0.2	Beta_2_ coronary and bronchial dilatation	5	5	

eNOS, Endothelial nitric oxide synthase; NE, norepinephrine; PHE, phentolamine; SB, salbutamol.

### PET imaging

Rest and adrenergic stress (NE and SB) myocardial blood flow index (MBFi) values were measured with [^11^C]acetate micro-PET to assess the coronary vasodilatation flow reserve as stress/rest MBFi ratio. Validation of [^11^C]acetate properties as a myocardial perfusion tracer has been confirmed recently [15],[23]. For the stress study, a constant infusion of NE or SB was initiated for 10 min. Adrenergic stress was induced using a constant infusion of NE or SB for 10 min. Five minutes later, a slow-bolus injection of 25 ± 9 MBq of [^11^C]acetate in 75-μL saline solution was administered using a syringe pump at a rate of 100 μL/min into the tail vein. PET imaging was performed with a 10.0-cm trans-axial, 12.7-cm axial field-of-view scanner (Inveon DPET, Siemens, Knoxville, TN, USA) with an isotropic spatial resolution of 1.4 mm. List-mode PET data were acquired for 5 min and binned into 18 dynamic frames of 12 × 10 s and 6 × 30 s. Dynamic images were reconstructed with 0.34 × 0.34 × 0.80 mm pixel size using 12 iterations of OSEM3D including corrections for detector dead-time, isotope decay, geometric efficiency, random coincidences, and system sensitivity, but not attenuation or scatter [24].

### Tracer kinetic modeling

From the reconstructed images, kinetic analysis of the myocardial time-activity data was performed semi-automatically using FlowQuant© v2.4 software (University of Ottawa Heart Institute, Ottawa, Ontario, Canada) as previously described [25],[26].

**Figure 1:**
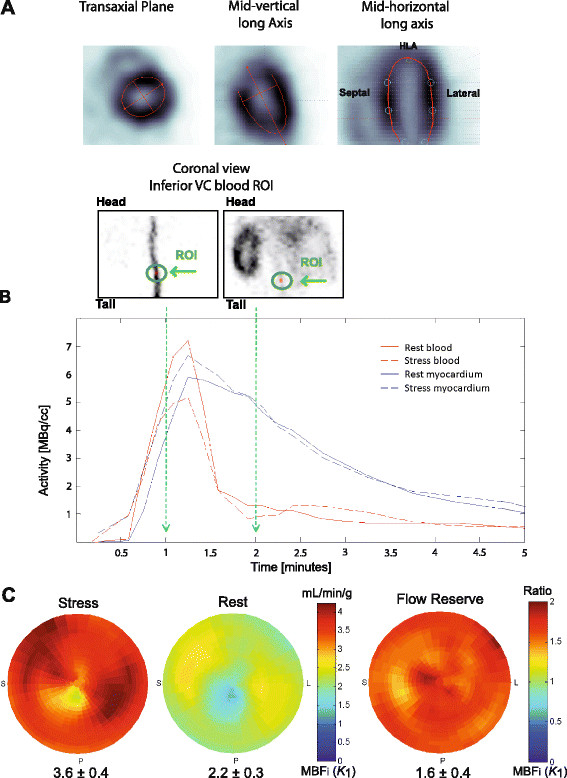
**Reorientation and sampling of myocardial ROI, metabolite-corrected blood input functions and myocardial TACs, and MBFi** ***K***_**1**_**polar-maps. (A)** Reorientation and sampling of the myocardial region of interest (ROI) in the transverse, vertical long-axis, and horizontal long-axis planes (FlowQuant© software). The image-derived input function (IDIF) from the inferior *vena cava* (VC) blood region (red) was created semi-automatically. **(B)** Metabolite-corrected blood input functions and myocardial TACs at rest and stress. **(C)** Myocardial blood flow index (MBFi) *K*_1_ polar-maps derived using the inferior VC blood input at Rest, low-dose salbutamol Stress, and stress/rest (Flow Reserve).

[^11^C]acetate blood time-activity curves (TACs) were obtained from an image-derived region-of-interest on the inferior *vena cava* (VC) using Inveon™ Research Workplace (IRW) software (Siemens; Knoxville, TN, USA). A 50% intensity contour-region-of-interest (ROI) was defined semi-automatically, on a 10-s frame with the highest VC activity (typically 30 to 40 s after injection), using a trans-axial slice 2 to 5 mm below the kidneys, avoiding activity spillover from surrounding organs during the dynamic scan (Figure 1A). The maximum activity in the VC region was measured at all time points, and the resulting blood TAC was shifted in time (+10 s) to match the initial upslope and peak activity of the arterial blood samples described below. This inferior VC image-derived input function (IDIF) was then used as input to a one-tissue-compartment model of [^11^C]acetate kinetics in the FlowQuant© program. The tracer kinetic model was fit to the left ventricular (LV) myocardium TAC data in each polar-map sector to estimate the tracer uptake rate (*K*_1_) as a MBFi [mL/min/g] and the tracer washout rate (*k*_2_) as an index of myocardial oxygen consumption (MVO_2_) [min^−1^] (Figure 1C) [17].

The stress/rest ratio of *k*_2_ values represents an index of the MVO_2_ stress response. The ratio of stress/rest *K*_1_ is an index of the MBF response (Figure 1C). A myocardial oxygen extraction fraction index (OEFi) was also estimated as the ratio of MVO_2_/MBFi. The LV-average polar-map values of stress/rest MBFi were also adjusted for changes in the RPP, reflecting alterations in cardiac demand and associated metabolic-dependent changes in flow [27]. The RPP-adjusted values were calculated as follows: [stress/rest MBFi]/[stress/rest RPP] = [stress MBFi/RPP]/[rest MBFi/RPP] and reported as sympathetic-stress myocardial flow reserve (sMFR). The specific changes in sMFR with NOS inhibition are reported as the nitric-oxide-mediated endothelial flow reserve (EFR_NOM_).

### Arterial blood sampling

Accuracy of the inferior VC IDIF described above was confirmed experimentally by simultaneous blood sampling from the carotid artery (standard (STD)) during [^11^C]acetate imaging at rest (*n* = 5) according to the following time sequence: 0, 10, 20, 30, 40, 45, 50, 55, 60, 70, 80, and 90 s and 2, 3, and 5 min, using a total blood volume of 0.5 ± 0.1 mL. The arterial blood samples were diluted in saline to a standard volume of 300 μL. The activity concentration (Bq/mL) was then measured using a calibrated well-counter (Packard Cobra II, Canberra, Meriden, CT, USA) and decay-corrected to the PET scan start time. The STD arterial TAC was used as input to the same tracer kinetic model described above for comparison of the MBFi values to those obtained using the inferior VC IDIF.

### Statistical analysis

Areas under the arterial blood curve and the derived MBFi values were compared between the VC and STD curves using a two-tailed paired *t*-test. Hemodynamic and MBF parameters were compared during SB-stress and PHE + NE-stress vs. NE control using F-tests and two-tailed unpaired *t*-tests. To assess physiologic changes in oxygen demand, RPP, stress/rest MBFi, sMFR (RPP-adjusted MBFi ratio), and MVO_2_ were compared between groups (PHE + NE-stress, SB-stress) using one-way ANOVA and two-tailed unpaired *t*-tests. Hemodynamic and MBFi parameters at rest were compared in the NOS-inhibition and eNOS-knockout groups vs. NE control using a one-way ANOVA. Adrenergic-stress MBFi was compared between control and NOS-inhibition groups using F-tests and *post hoc* two-tailed unpaired *t*-tests. All data were expressed as mean ± standard deviation (SD). A *p* value less than 0.05 was considered statistically significant.

## Results

### Arterial blood input function validation

**Figure 2:**
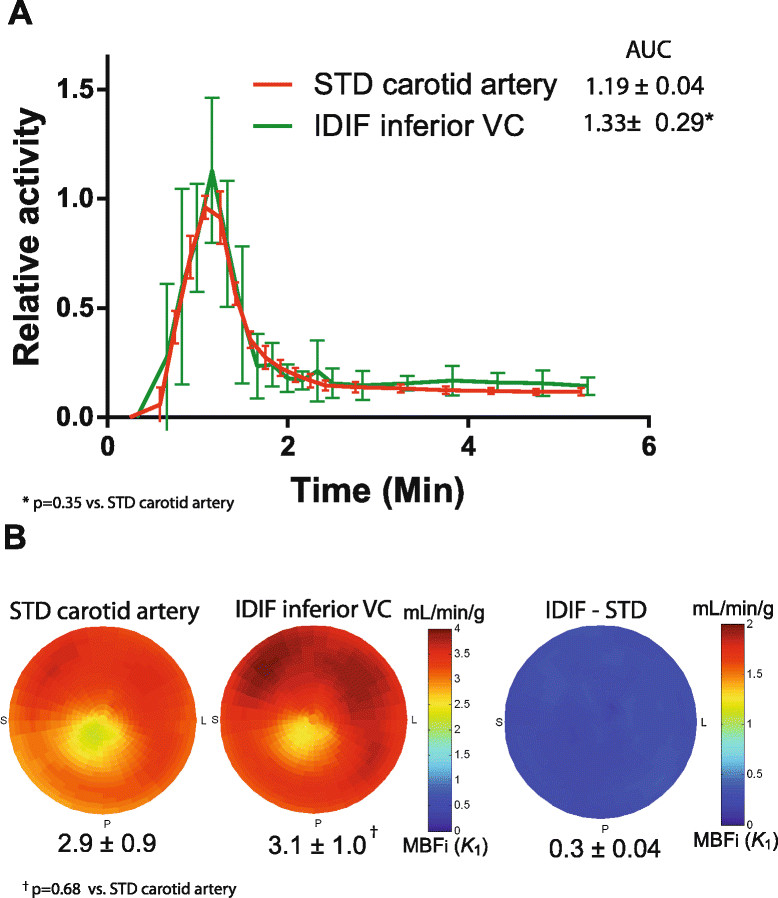
**Average blood input functions following [**^**11**^**C]acetate injection and MBFi polar-maps using carotid artery STD and inferior VC blood inputs. (A)** Average blood input functions following [^11^C]acetate injection in normal control mice (*n* = 5). Curves are normalized relative to the maximum activity of the carotid artery reference standard (STD) samples. **(B)** Myocardial blood flow index (MBFi) polar-maps derived using the carotid artery STD and inferior *vena cava* (VC) blood input curves. There were no significant differences in blood input areas-under-the-curve (AUCs) or MBFi values.

The carotid artery STD and the inferior VC blood input curves are shown in Figure 2. The time-shifted (10-s delay) IDIF obtained from the inferior VC region matched the blood curve sampled from the carotid artery, demonstrating similar shapes and no significant difference in the area-under-the-curve (*p* = 0.35). The corresponding MBFi values were not significantly different (3.1 vs. 2.9 mL/min/g, *p* = 0.68). Regional variation in the difference between the two MBFi polar-maps was also low (13% coefficient-of-variation across polar-map sectors), suggesting that no regional bias was induced by using the time-shifted inferior VC blood input.

### Selective beta-adrenergic MBF response

**Figure 3:**
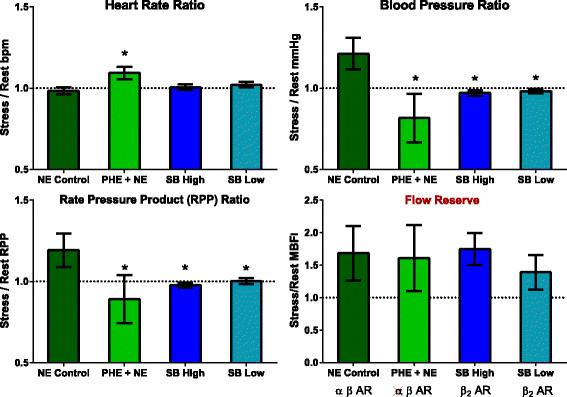
**Hemodynamics and myocardial blood flow index (MBFi) response to adrenergic stress agents in healthy normal mice.** NE norepinephrine 3.2 μg/kg/min, PHE phentolamine pretreatment, SB salbutamol High 1.0 μg/kg/min, SB salbutamol Low 0.2 μg/kg/min. NE-stress (green), SB-stress (blue). *p < 0.05 vs. NE Control.

The effects of β-specific and β_2_-selective adrenergic stimulation on MBF are shown in Figure 3. In contrast to the NE-stress response (with or without PHE), the proposed β_2_-selective agonist SB had no significant effect on the hemodynamic parameters (Table 2). A dose-dependent increase of 39% to 75% in stress vs. rest MBFi was observed (*p* < 0.0001), suggesting a cardiac-specific effect of SB at both doses (Table 3). There was no significant effect of body weight on the measured MBFi values between groups (ANOVA *p* = 0.08).

**Table 2:** **Hemodynamic response to NE- and SB-stress**

Group	Stressor dose (μg/kg/min)	Mouse weight (g)	Rest			Stress		
			Heart rate (bpm)	SBP (mmHg)	RPP (bpm∙mmHg)	Heart rate (bpm)	SBP (mmHg)	RPP (bpm∙mmHg)
Normal	NE 3.2	25 ± 3	409 ± 29	74 ± 12	30,018 ± 4,699	402 ± 25	89 ± 11	35,639 ± 4,557
	SB 0.2	27 ± 1	423 ± 21	88 ± 12	37,430 ± 5,850	433 ± 20	87 ± 14	37,593 ± 6,140
	SB 1.0	26 ± 1	432 ± 13	87 ± 13	37,729 ± 6,275	434 ± 11	85 ± 14	36,884 ± 6,587
	PHE + NE 3.2	25 ± 3	435 ± 32	95 ± 17	40,967 ± 6,151	476 ± 36*	77 ± 17	36,390 ± 7,695
eNOS knockout	NE 3.2	27 ± 6	355 ± 41*^,^**	107 ± 19*^,^**	38,516 ± 11,584	373 ± 42*^,^**	114 ± 17*^,^**	43,018 ± 11,214
NOS inhibition	NE 3.2	28 ± 3	454 ± 22*	79 ± 20	36,024 ± 10,195	453 ± 24*	85 ± 22	38,511 ± 10,921
	SB 0.2	31 ± 3	404 ± 44	90 ± 10	36,877 ± 5,278	409 ± 43	89 ± 8	37,021 ± 5,305
	SB 1.0	30 ± 2	408 ± 32	91 ± 11	37,247 ± 5,613	406 ± 30	91 ± 12	37,072 ± 6,122

bpm, heart beats per minute; SBP, systolic blood pressure; RPP, heart rate × systolic blood pressure product; eNOS, endothelial nitric oxide synthase; NE, norepinephrine; SB, salbutamol. NOS inhibition = L-NAME pretreated. Values are mean ± standard deviation. *p < 0.05 vs. normal NE. **p < 0.05 vs. NOS-inhibition NE.

**Table 3:** **MBFi (** ***K***_**1**_ **), MVO**_**2**_ **(** ***k***_**2**_ **), and RPP responses to NE- and SB-stress**

Group	Stressor dose (μg/kg/min)	MBFi ratio (stress/rest)	MVO_2_ratio (stress/rest)	OEFi ratio (stress/rest)	RPP ratio (stress/rest)	sMFR (RPP-adjusted)
Normal	NE 3.2	1.68 ± 0.42	1.58 ± 0.28	0.99 ± 0.29	1.19 ± 0.10	*1.39 ± 0.35 (25%)*
	SB 0.2	*1.39 ± 0.27*	1.37 ± 0.15	1.00 ± 0.10	1.00 ± 0.02*	*1.39 ± 0.27 (19%)*
	SB 1.0	1.75 ± 0.25***	1.73 ± 0.23***	1.00 ± 0.14	0.98 ± 0.02*	1.78 ± 0.25*** (14%)
	PHE + NE 3.2	1.61 ± 0.51	1.95 ± 0.45	1.27 ± 0.35	0.89 ± 0.15*	1.81 ± 0.57 (32%)
eNOS knockout	NE 3.2	1.12 ± 0.23*	1.57 ± 0.34	1.46 ± 0.50	1.13 ± 0.04	0.99 ± 0.20* (20%)
NOS inhibition	NE 3.2	1.03 ± 0.23*	1.35 ± 0.20	1.35 ± 0.29	1.07 ± 0.02*	0.96 ± 0.22* (23%)
	SB 0.2	0.99 ± 0.15*^,^**	1.17 ± 0.08*^,^**	1.20 ± 0.12**	1.00 ± 0.03*	0.98 ± 0.15*^,^** *(15%)*
	SB 1.0	1.38 ± 0.17**^,^***	1.58 ± 0.37***	1.16 ± 0.29	0.99 ± 0.02*	1.39 ± 0.17**^,^*** (12%)

MBFi, myocardial blood flow index; MVO_2_, myocardial oxygen consumption; OEFi = MVO_2_/MBFi, oxygen extraction fraction index; RPP, heart rate × systolic blood pressure product; eNOS, endothelial nitric oxide synthase; NE, norepinephrine; SB, salbutamol. Sympathetic-stress myocardial flow reserve (sMFR) RPP-adjusted = MBFi ratio/RPP ratio; NOS inhibition = L-NAME pretreated. Nitric-oxide-mediated endothelial flow reserve (EFR_NOM_) values shown in *italics*. Values are mean ±standard deviation (coefficient of variation × 100%). *p < 0.05 vs. normal NE. **p < 0.05 vs. normal SB with same dose. ***p < 0.05 vs. low-dose SB (0.2 μg/kg/min).

The RPP-adjusted sMFR in the PHE + NE group (1.81) was increased by 12% compared to the unadjusted MBFi ratio, resulting in values very close to the high-dose SB results (1.78). Conversely, sMFR in the NE control group (1.39) was decreased by 17% compared to the unadjusted MBFi ratio, resulting in values equivalent to the low-dose SB group (1.39). Therefore, it appears that only SB, through selective activation of the β_2_-adrenergic receptors, was able to produce a myocardial flow response without significantly changing the systemic hemodynamic parameters, as opposed to NE with or without PHE pretreatment.

### NO-mediated myocardial blood flow response

**Figure 4:**
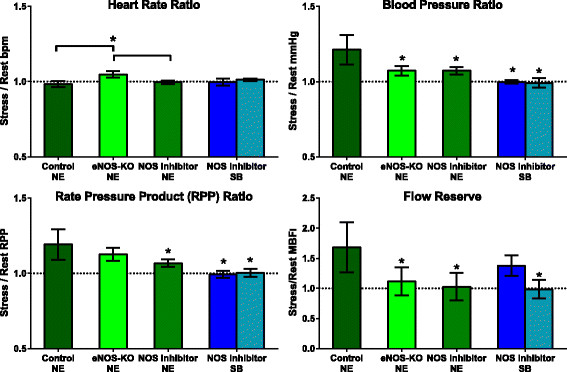
**Hemodynamics and myocardial blood flow index (MBFi) response to adrenergic stress agents norepinephrine (NE) and salbutamol (SB) with and without NOS inhibition.** Normal controls are compared to NOS inhibition (L-NAME pretreatment), and endothelial nitric oxide synthase knockout mice (eNOS knockout). NE-stress (green), SB-stress (high dose, dark blue; low dose, light blue). *p < 0.05 vs. NE Control.

The specific NO-mediated (i.e. endothelial-dependent) component of the adrenergic-stress MBFi responses is shown in Figure 4 and Table 3. Despite small hypertensive changes, the resting MBFi values in the eNOS-knockout group (3.3 ± 0.6) were similar to the normal controls (3.4 ± 1.3, *p* = 0.80) and NOS-inhibition groups (4.0 ± 1.3, *p* = 0.24).

**Figure 5:**
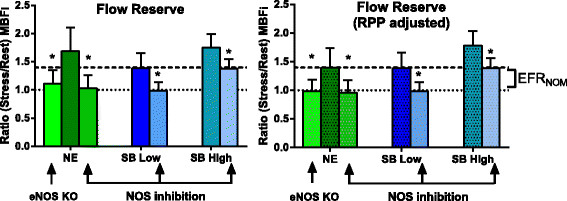
**Flow reserve response to β-adrenergic vasodilator stressors, NOS inhibition, and eNOS knockout.** NE norepinephrine 3.2 μg/kg/min, SB High salbutamol 1.0 μg/kg/min, SB Low salbutamol 0.2 μg/kg/min. High-dose SB flow reserve is the sum of NO-mediated endothelial (EFR_NOM_) and non-NO-mediated microvascular flow responses. NE-stress (green), SB-stress (blue). *p < 0.05 NOS inhibition vs. same stressor without NOS inhibition.

With NE-stress, the NOS-inhibition and eNOS-knockout groups had similar endothelial-dependent reductions in MBFi ratio compared to the NE control (39% and 33%, respectively), with values close to unity (0.96 and 0.99) confirming that the RPP-adjusted NE-stress response was NO-mediated, and therefore predominantly reflected the coronary-specific EFR_NOM_ (Figure 5).

The high-dose and low-dose SB groups under NOS inhibition showed similar decreases in MBFi ratio (−0.37 and −0.4, respectively) and MVO_2_ ratio (−0.2 and −0.15, respectively) compared to normal mice at the same dose. However, only the low-dose SB group had a MBFi ratio close to unity (0.99), with or without RPP adjustment. Similar to NE-stress in the NOS-inhibition and eNOS-knockout groups above, the low-dose SB flow response was completely blocked therefore reflecting EFR_NOM_ (Figure 5). In contrast, the high-dose SB group maintained a MBF response significantly greater than unity (1.39), suggesting that the β_2_-adrenergic agonist is only partially endothelial-dependent and NO-mediated at this dose, with the remaining increase being dependent on non-NO-mediated mechanisms. With SB-stress, NOS-inhibition mice showed no significant hemodynamic changes vs. normal controls (Table 3). These results demonstrate that the high-dose and low-dose SB-stress/rest flow responses were partially and completely NO-mediated, respectively. Low-dose SB appeared to specifically reflect the NO-mediated endothelium-dependent vasodilatation (i.e., EFR_NOM_).

## Discussion

To our knowledge, this is the first study to demonstrate the use of SB-stress to selectively measure the NO-mediated endothelial blood flow response using non-invasive micro-PET imaging. Our findings shed new light on the mechanisms and effects of coronary β-specific vasodilatation *in vivo*. Previous results have only been obtainable using invasive or *ex vivo* methodologies, which are not feasible or optimal for application in mouse models of endothelial dysfunction. Low-dose SB-stress PET imaging appears to be well suited to measure coronary-specific NO-mediated endothelial vasoreactivity, e.g., for preclinical investigation of targeted therapies to improve NO bioavailability and contractile function of the vascular endothelium [20].

### Beta-adrenergic myocardial blood flow response

**Figure 6:**
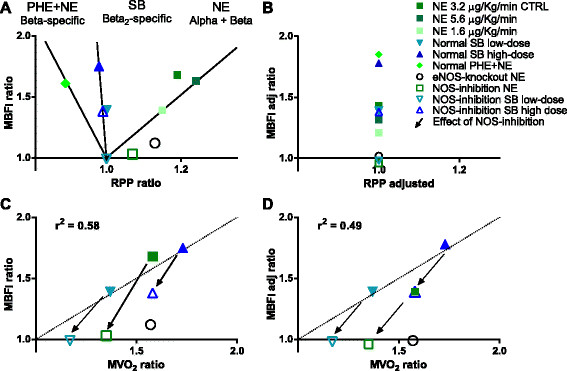
**NOS-inhibition effects on myocardial blood flow index response (MBFi ratio) to adrenergic stress agents.** Norepinephrine (NE) 1.6, 3.2, and 5.6 μg/kg/min and salbutamol (SB) low-dose 0.2 μg/kg/min and high-dose 1.0 μg/kg/min. **(A)** MBFi vs. RPP response (stress/rest), **(B)** MBFi adjusted to the mean RPP-ratio (MBFi_adj_ ratio), **(C)** MBFi vs. oxygen metabolism response (stress/rest MVO_2_ ratio), and **(D)** adjusted MBFi vs MVO_2_ ratio. After adjusting for the RPP-effects on MBFi, NOS inhibition results in proportional reductions in the MBFi and MVO_2_ responses to adrenergic stress (arrows shown in **(D)**).

In the present study, the catecholamine NE, a potent α_1_, α_2_, moderate β_1_, and potent β_2_ agonist, was selected initially for its well-established role in the human CPT response [28]. Similar to dobutamine, the positive chronotropic and inotropic effects of NE on heart rate and blood pressure were observed [29]. Control of coronary blood flow during exercise is achieved by close coupling of the NE blood level and myocardial oxygen consumption [4],[30]. Indeed, a good correlation (*r*^2^ = 0.65) was observed between the MFR and MVO_2_ indices across all groups (Figure 6), equivalent to that observed in previous human studies [31]. The selected NE control dose was targeted for measurement of the maximal sMFR response associated with endothelial-dependent activation *without* increasing the heart rate. Adjustment of the MBF response for changes in the stress/rest RPP ratio was performed to account for any change in oxygen demand induced with NE-stress, to obtain a specific measure of the EFR_NOM_[32]. The known relationship between oxygen demand and the metabolic-dependent signaling pathways for vasodilatation would otherwise be confounding factors, precluding specific measurement of the endothelial-dependent vasodilatation.

After RPP adjustment, the NE control values of sMFR in the normal mice were equal to the low-dose SB values (1.4-fold increase), reflecting a similar level of endothelium-dependent (and NO-mediated) stimulation. In the high-dose SB and PHE + NE groups, the RPP-adjusted MBFi response (1.8-fold increase) was higher compared to the NE control and low-dose SB, suggesting added effects from other mechanisms of vasodilatation. With NE- and SB-stress (high- or low-dose) in the normal control mice, there was a matched increase in MBFi and MVO_2_, i.e., OEFi ratio values were very close to 1.0 confirming the close coupling of metabolism and flow under normal conditions. Under SB-stress, the observed increase in MVO_2_ without any change in the RPP is somewhat unexpected but may be related to secondary metabolic effects resulting from activation of β_2_-receptors on the cardiac myocytes.

### NO-mediated myocardial blood flow response

Administration of L-NAME, a NOS inhibitor, leads to a decrease in NO bioavailability and creates an imbalance between vasodilatation and vasoconstriction, resulting in a trend towards increased peripheral blood pressure (Table 2) from unopposed α-adrenergic tone [22].

At rest, MBFi was not significantly changed in the NOS-inhibition or eNOS-knockout groups, consistent with previous literature [33]. The NOS-inhibition and eNOS-knockout groups showed a complete inhibition of the sMFR response to NE-stress. With intravenous SB, our non-invasive EFR_NOM_ results using [^11^C]acetate micro-PET were similar to previous invasive intracoronary infusion studies showing that a hyperemic response was partially or totally blocked in the presence of a NOS inhibitor [3],[12].

With high-dose and low-dose SB-stress, the same decrease in sMFR under NOS inhibition was observed compared to normal control mice without NOS inhibition, suggesting a similar magnitude of NO-mediated vasodilatation (EFR_NOM_ approximately 1.4-fold increase). Low-dose SB resulted in an exclusively NO-mediated endothelium-dependent effect, where MBFi under NOS inhibition was 100% inhibited with or without RPP adjustment (therefore reflecting EFR_NOM_). This is in contrast to high-dose SB, where only a 50% reduction of the total sMFR was observed with NOS inhibition. This observation may be explained by the presence of β_2_-adrenergic receptors on the coronary vascular endothelium *and* smooth muscle cells [34]. High-dose SB may activate both components of the coronary vessel wall, as well as other non-NO-mediated signaling mechanisms, whereas low-dose SB seems to produce a coronary-specific and endothelium-dependent NO-mediated activation [12].

Compared to the complete NOS inhibition of the MBF response with low-dose salbutamol, there was only a partial reduction in the MVO_2_ response compared to controls, corresponding to a significantly increased OEFi ratio (1.2 vs. 1.0; *p* < 0.05). A similar trend was observed in the other NOS-inhibition and eNOS-knockout groups, but the changes in OEFi ratio did not reach statistical significance, possibly due to the small sample size. These changes in the OEFi ratio suggest an uncoupling of metabolism and blood flow, as might be expected in these animal models of endothelial dysfunction. Further studies are needed to fully investigate the physiologic mechanism(s) of these observed changes in oxidative metabolism.

These results demonstrate a strong potential for the proposed low-dose SB-stress method to be translated to humans as a coronary adrenergic stress test of NO-mediated endothelial function (Figure 5). For future translation to clinical PET imaging studies, the clinical safety profile of intravenous SB is well-established in the treatment of acute asthma, using doses that are the same as those proposed in the present study to measure the endothelial-dependent flow reserve (low-dose SB = 0.2 μg/kg/min × 10 min) [35].

### Kinetic modeling analysis

As a well known metabolic radiotracer for oxygen consumption, [^11^C]acetate has also been validated as a radiotracer for MBF imaging in humans and rats. There is good correlation between MBF values obtained with compartment modeling of [^11^C]acetate and [^15^O]water or [^13^ N]ammonia [23]. In the single-tissue compartment model, the myocardial efflux of [^11^C]CO_2_ and the clearance of other labeled metabolites are typically combined in a single rate constant (*k*_2_) reflecting the rate of oxidative metabolism. An average blood metabolite correction has been used to obtain an accurate arterial input function providing MBF estimates in healthy and hypertrophic cardiomyopathy subjects [23]. A potential limitation of this method is the non-linear correlation between *K*_1_ and MBF [17]; further investigation will be needed to evaluate the quantitative accuracy in mice. In the present study, the use of stress/rest *ratio* responses should help to minimize the effects of any potential bias in the *K*_1_ index of MBF.

While the use of [^11^C]acetate for MBF quantification has been validated at rest and during adenosine stress in humans [17], it has not been separately validated in mice or under catecholamine stress. In humans, exercise and dobutamine stress have been used with [^11^C]acetate to assess MBF and oxidative metabolism reserve through stimulation of the sympathetic system and through adrenergic receptor activation [36],[37]. In the present study, we used the tracer uptake rate constant (*K*_1_) as an index of MBF to assess the response to adrenergic stimulation using NE and SB. Estimation of absolute MBF derived from the [^11^C]acetate *K*_1_ values in mice would require the use of a species-specific tracer extraction function, which has not been published to our knowledge. An oxidative stress response to catecholamines might affect the tracer *k*_2_ values reflecting the rate of oxidative metabolism and hence changes in the oxygen extraction fraction (OEF) under NOS and α-adrenergic inhibition as indicated in Table 3. Conversely, oxidative stress seems unlikely to affect the initial tracer uptake rate (*K*_1_) which reflects diffusion and transport across the capillary and cell membranes [37].

Partial-volume and spillover effects in cardiac micro-PET imaging have been a challenge in the past for tracer kinetic modeling analysis. The present study included validation of the image-derived input function (Figure 1) and geometric spillover correction to obtain accurate quantitative data, similar to previous studies [38]. A 10-s shift of the inferior VC blood curve was included to account for the venous to arterial transport delay, whereas dispersion was not observed using the controlled slow-bolus tracer injection. Similar MBFi values were obtained using both the inferior VC and STD arterial sampled blood input curves, confirming the accuracy of the image-derived VC input curve [24].

## Conclusions

Low-dose salbutamol β_2_-adrenergic receptor stimulation induces coronary-specific and NO-mediated endothelium-dependent vasodilatation. Salbutamol-stress [^11^C]acetate micro-PET imaging of EFR_NOM_ may be suitable for assessment of coronary endothelial dysfunction in small animal models of disease and evaluation of new therapies. The EFR_NOM_ response to low-dose SB-stress was exclusively NO-mediated, and the method may be translated easily to human studies.

## Authors’ original submitted files for images

Below are the links to the authors’ original submitted files for images. Authors’ original file for figure 1 Authors’ original file for figure 2 Authors’ original file for figure 3 Authors’ original file for figure 4 Authors’ original file for figure 5 Authors’ original file for figure 6
