# Supplementary figures and images for: β2-adrenergic stress evaluation of coronary endothelial-dependent vasodilator function in mice using 11C-acetate micro-PET imaging of myocardial blood flow and oxidative metabolism
Source: EJNMMI Res. 2014 Dec 16;4:68. doi: 10.1186/s13550-014-0068-9 (PMC4293492; doi:10.1186/s13550-014-0068-9)

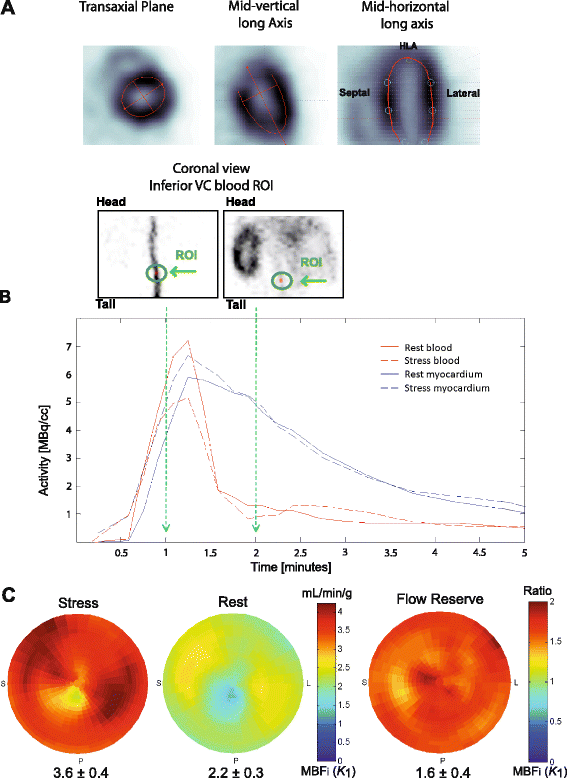

Supplement: Supplementary file 1 — Authors’ original file for figure 1 [file 13550_2014_68_MOESM1_ESM.gif]

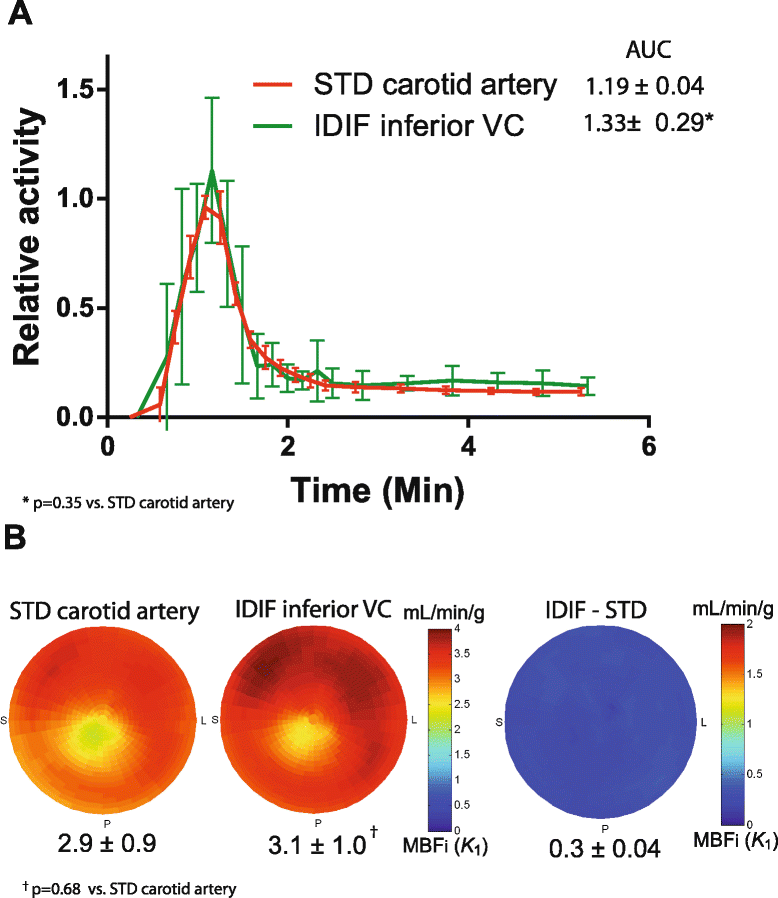

Supplement: Supplementary file 2 — Authors’ original file for figure 2 [file 13550_2014_68_MOESM2_ESM.gif]

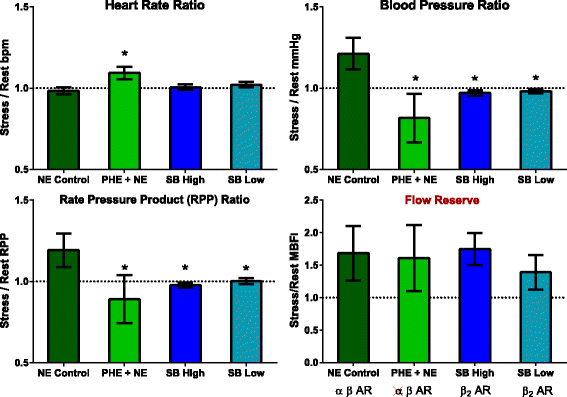

Supplement: Supplementary file 3 — Authors’ original file for figure 3 [file 13550_2014_68_MOESM3_ESM.gif]

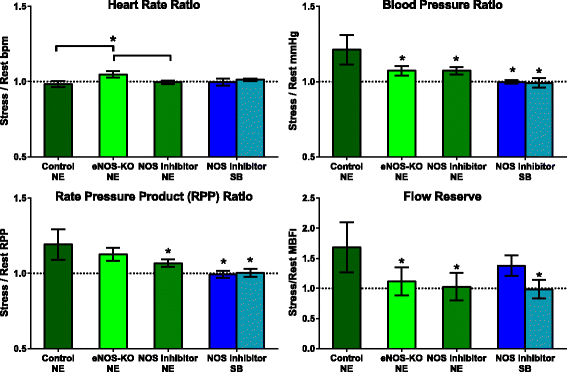

Supplement: Supplementary file 4 — Authors’ original file for figure 4 [file 13550_2014_68_MOESM4_ESM.gif]

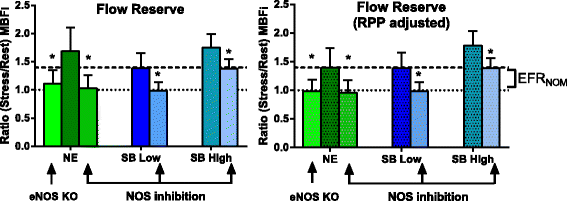

Supplement: Supplementary file 5 — Authors’ original file for figure 5 [file 13550_2014_68_MOESM5_ESM.gif]

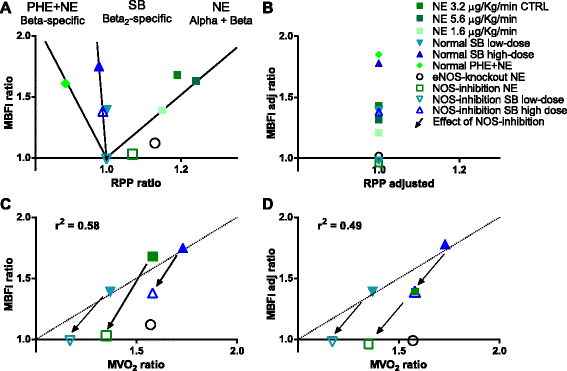

Supplement: Supplementary file 6 — Authors’ original file for figure 6 [file 13550_2014_68_MOESM6_ESM.gif]
